# Berberine Inhibits MDA-MB-231 Cells as an Agonist of G Protein-Coupled Estrogen Receptor 1

**DOI:** 10.3390/ijms222111466

**Published:** 2021-10-24

**Authors:** Miaomiao Qi, Xiang Liu, Ying Zhou, Haoyu Wang, Ying Zhao, Jing Ren, Jin Xiang

**Affiliations:** 1 Key Laboratory of Combinatorial Biosynthesis and Drug Discovery, Ministry of Education, School of Pharmaceutical Sciences, Wuhan University, Wuhan 430071, China; qmmwhu@126.com (M.Q.); liuxiang19941224@whu.edu.cn (X.L.); why616@whu.edu.cn (H.W.); zying1305@163.com (Y.Z.); rj1994@whu.edu.cn (J.R.); 2 Research Center for Medicine and Structural Biology of Wuhan University, Wuhan University, Wuhan 430071, China; zhouying2004@whu.edu.cn

**Keywords:** berberine, binding, co-localization, GPER1, MAP1LC3, NF-κB

## Abstract

G protein-coupled estrogen receptor 1 (GPER1) is a potential therapeutic target for treating triple-negative breast cancers (TNBC). However, modulators for GPER1 that can be used to treat TNBC have not appeared. Berberine (BBR) is a bioactive isoquinoline alkaloid with high oral safety. In recent years, BBR has shown an inhibitory effect on TNBC tumors such as MDA-MB-231, but the molecular target remains unclear, which hinders related clinical research. Our work proved that BBR is a modulator of GPER1 that can inhibit cell viability, migration, and autophagy of MDA-MB-231 cells. The inhibitory effect of BBR on MDA-MB-231 cells has a dependence on estrogen levels. Although BBR promoted the proteasome, which is a major factor in the degradation of GPER1, it could still induce the protein level of GPER1. Correspondingly, the transcription of cellular communication network factor 2 (CCN2) was promoted. BBR could bind to GPER1 directly and change the secondary structure of GPER1, as in the case of 17β-estradiol (E2). In addition, BBR induced not only a high degree of co-localization of GPER1 and microtubule-associated protein 1 light chain 3 (MAP1LC3), but also the accumulation of sequestosome 1 (SQSTM1/p62) by the inhibition of the nuclear translocation of the nuclear factor-kappa B (NF-κB) subunit (RELA/p65), which indicates NF-κB inhibition and anti-cancer effects. This result proved that the promotional effect of BBR on the GPER1/NF-κB pathway was closely related to its inhibitory effect on autophagy, which may serve as a new mechanism by which to explain the inhibitory effect of BBR on MDA-MB-231 cells and expand our understanding of the function of both BBR and GPER1.

## 1. Introduction

Triple-negative breast cancer (TNBC) is a breast cancer in which estrogen receptor 1 (ESR1), progesterone receptor (PR), and human epidermal growth factor receptor 2 (HER-2) are all negative [1]. Patients with TNBC account for approximately 10–15% of all breast cancer patients [2]. TNBC has the characteristics of high mitosis rate, high lymphocyte infiltration, high grade, and large tumor size. The main way to treat TNBC is still chemotherapy and radiotherapy. In all five routinely used molecular subtypes (Luminal A-like, Luminal B/HER2 negative-like, Luminal B/HER2 positive-like, HER2-type, TNBC), TNBC tumors presented the poorest prognosis with a local recurrence rate (LCR) of 89.6%, disease-free survival (DFS) of 69.1%, distant disease-free survival (DDFS) of 72.2%, observed overall survival (OS) of 78.5%, and relative overall survival (ROS) of 80.1% at five years (95% confidence interval) [3]. It is important to find new treatments for TNBC [4].

BBR is an isoquinoline alkaloid that has been used in the treatment of gastroenteritis and dysentery for a long time with high oral safety [5]. In recent years, berberine has been shown to inhibit a variety of cancers in cell and animal research [6,7,8,9]. The antitumor cellular effects of BBR related to breast cancer mainly include (1) inducing apoptosis of MCF-7 by promoting ROS formation, overexpressing TP53 and WAF1/p21, and down-regulating BCL2; (2) inhibiting the metastasis of MDA-MB-231 cells by down-regulating PI3K/AKT, NF-κB, AP-1, and matrix metallopeptidase MMP2/9; (3) affecting the autophagy and growth of breast cancer cells by up-regulating beclin-1, down-regulating BCL2, and inhibiting mTOR [10,11,12,13].

Although BBR has shown multiple cellular effects against breast cancer, including the highly aggressive TNBC cell line MDA-MB-231 as above, the exact mechanism remains unclear. The correlation between cell proliferation, migration, and autophagy increases the difficulty of determining the specific target of BBR against breast cancers. To reveal the mechanism of BBR’s inhibition on MDA-MB-231 cells, we performed a series of studies. MTT showed that the inhibitory effect of BBR on MDA-MB-231 cells was much stronger than those on two other TNBCs (MDA-MB-436, and MDA-MB-468). Therefore, MDA-MB-231 cells were taken as the main research object in this study.

Label-free MS revealed that BBR could affect both autophagy and estrogen signaling pathways in MDA-MB-231. G protein-coupled estrogen receptor 1 (GPER1) is a new type of estrogen receptor that was reported at the end of the 20th century. In contrast to ESRs, GPER1 is widely expressed in breast cancers including TNBCs, which mediates the rapid signal response of estrogen and is related to the drug resistance of tamoxifen and other drugs [14]. GPER1 is closely related to the structural deformation of breast glands, which is one important sign of the deterioration of breast tissue. In addition, estrogen can intervene in autophagy and the growth of breast cancer cells via GPER1, which makes GPER1 a potential therapeutic target to treat TNBC [15,16]. Cellular communication network factor 2 (CCN2) is a target gene of GPER1 [17]. NF-κB is a key link between estrogen receptors and autophagy [18,19,20,21]. The 20S proteasome is a major site for the degradation of both intracellular and plasma membrane-derived GPER1 [22]. Microtubule-associated protein 1 light chain 3 (MAP1LC3) and sequestosome 1 (SQSTM1/p62) are protein markers most used to characterize autophagy. Here, we performed a series of studies to analyze the effect of BBR on these molecules in MDA-MB-231 cells, tested the activity of 20S proteasome, and studied the interaction between BBR and GPER1. The results revealed the molecular mechanism underlying the inhibitory effect of BBR on MDA-MB-231 cells, which may serve as a new mechanism to explain the inhibitory effect of BBR on MDA-MB-231 cells.

## 2. Results

### 2.1. Both Estrogen and Autophagy May Be Involved in Anti-Cancer Effects of BBR

**Figure 1:**
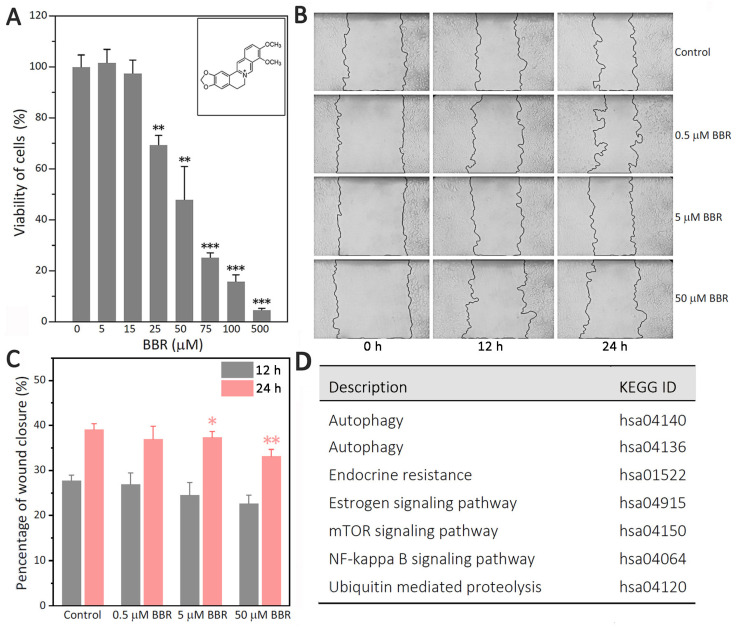
BBR inhibits MDA-MB-231 cells by affecting both autophagy and the estrogen signaling pathway. (**A**) MDA-MB-231 cells were treated with 0.1% DMSO as a vehicle control. The data are presented as mean ± SEM; *n* = 5. *p* < 0.01 (**), *p* < 0.001 (***). (**B**) The sizes of the scratched areas were observed at 0, 12, and 24 h. Representative images of each group are shown. (**C**) The percentages of wound closure are presented as mean ± SEM; *n* = 5. *p* < 0.05 (*), *p* < 0.01 (**). (**D**) The path analysis revealed that BBR could affect autophagy, estrogen effect, and some pathways closely related to them.

This experiment analyzed the effect of BBR on the viability of MDA-MB-231 cells. As shown in Figure 1A, the viability of MDA-MB-231 cells was significantly inhibited by BBR at concentrations of 25 μM or higher. The inhibition effect of BBR was positively correlated with the concentration of BBR. The half-maximal inhibitory concentration (IC_50_) of BBR was 46.3 μM, which was lower than that of MDA-MB-468 cells (IC_50_ = 78.5 μM). In addition, BBR showed no inhibition on MDA-MB-436 cells in the concentration range below 100 μM (Supplementary Figure S1).

To study the inhibitory effect of BBR on the migration of MDA-MB-231 cells, we treated cells with 0.1% DMSO, 0.5, 5, and 50 μM BBR after we scratched the cell layer. Then, we observed the migration of the cells in each group at 0, 12, and 24 h (Figure 1B). Compared with the control group, the percentage of wound closure were significantly reduced after 24-h treatment with 5 μM BBR (*p* < 0.05) and 50 μM BBR (*p* < 0.01) (Figure 1C).

To explore the key points of BBR when it acted on MDA-MB-231 cells, the proteins of 50 μM BBR-treated group were compared with that of control group by label-free MS. There were about 4071 proteins that could be extracted as the original data. After pretreatment, 4052 proteins were retained (Supplementary Table S2). As shown in Supplementary Table S3, there were a total of 787 differentially expressed proteins, in which 486 proteins increased and 301 proteins decreased. The path analysis results (Supplementary Table S4) revealed that BBR could affect estrogen signal and that some pathways were closely related, such as NF-κB, autophagy and ubiquitin-mediated proteolysis (Figure 1D).

### 2.2. BBR Treatment Induced GPER1

*CCN2* is a target gene of GPER1. NF-κB is at the downstream of GPER1 in MDA-MB-231. To further clarify how BBR influences estrogen signaling in MDA-MB-231 cells, the mRNA and protein levels of GPER1, CCN2, and RELA were observed after 1- and 4-h treatment with BBR.

**Figure 2:**
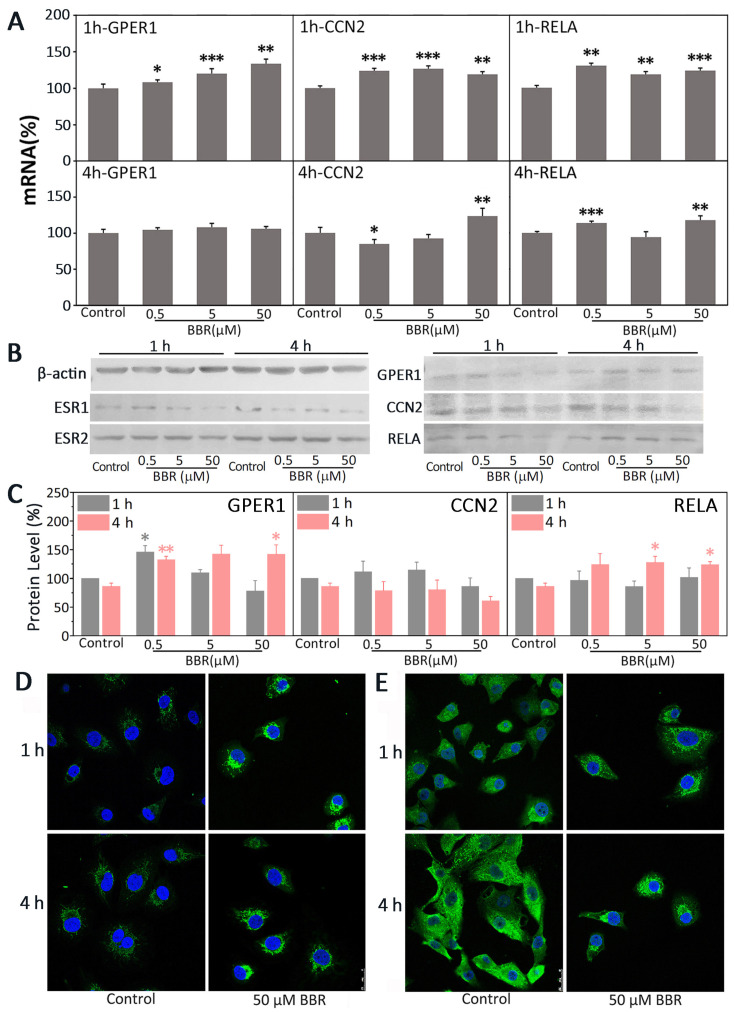
BBR upregulated GPER1 in MDA-MB-231 cells. MDA-MB-231 cells were treated with 0.1% DMSO, 0.5 μM BBR, 5 μM BBR, and 50 μM BBR. (**A**) The mRNA expression of both CCN2 and RELA was detected by RT-PCR. GAPDH was set as the reference. The data are presented as mean ± SEM; *n* = 5. *p* < 0.05 (*), *p* < 0.01 (**), *p* < 0.001 (***). (**B**) The protein expression of CCN2, RELA, GPER1, ESR1, and ESR2 in MDA-MB-231 cells was detected by Western blot. β-actin was set as the reference. (**C**) The protein expression levels of GPER1, CCN2, and RELA were calculated relative to the expression of β-actin, *n* = 3. *p* < 0.05 (*), *p* < 0.01 (**). (**D**) The nucleus and GPER1 (green) were marked with fluorescent dyes. (**E**) The nucleus and RELA (green) were marked with fluorescent dyes.

As shown in Figure 2A, 1-h treatment with BBR (0.5, 5, 50 μM) could promote the transcription of *GPER1*, *CCN2*, and *RELA* significantly. After 4 h of treatment, a high concentration of BBR (50 μM) still significantly promoted the transcription of both *CCN2* and *RELA* (*p* < 0.01). However, the promotional effect of BBR on the transcription of *GPER1* was no longer distinct (*p* > 0.05).

As shown in Figure 2B,C, it is clear that the protein level of GPER1 was increased significantly with 0.5 μM BBR after 1- or 4-h treatment (1 h, *p* < 0.05; 4 h, *p* < 0.01). The protein level of GPER1 was also distinctly promoted by 50 μM BBR after 4 h of treatment (*p* < 0.05). The protein level of RELA was promoted after 4-h treatment with 5 and 50 μM BBR (*p* < 0.05). However, the effects of BBR on the protein level of CCN2 was not distinct (*p* > 0.05). In addition, the expression of both ESR1 and ESR2 could also been observed. Compared with the 1-h results, the protein levels of GPER1, CCN2, and RELA had no significant changes in all the 4-h treatments (*p* > 0.05). As shown in Figure 2D, the fluorescence signal of GPER1 exhibited an increase after 1- or 4-h treatment with 50 μM BBR. Although the effect of 50 μM BBR on the protein level of RELA by immunostaining was undefined, the inhibitory effect of BBR on the nuclear translocation of RELA was clear (Figure 2E).

### 2.3. BBR Is an Agonist of GPER1

**Figure 3:**
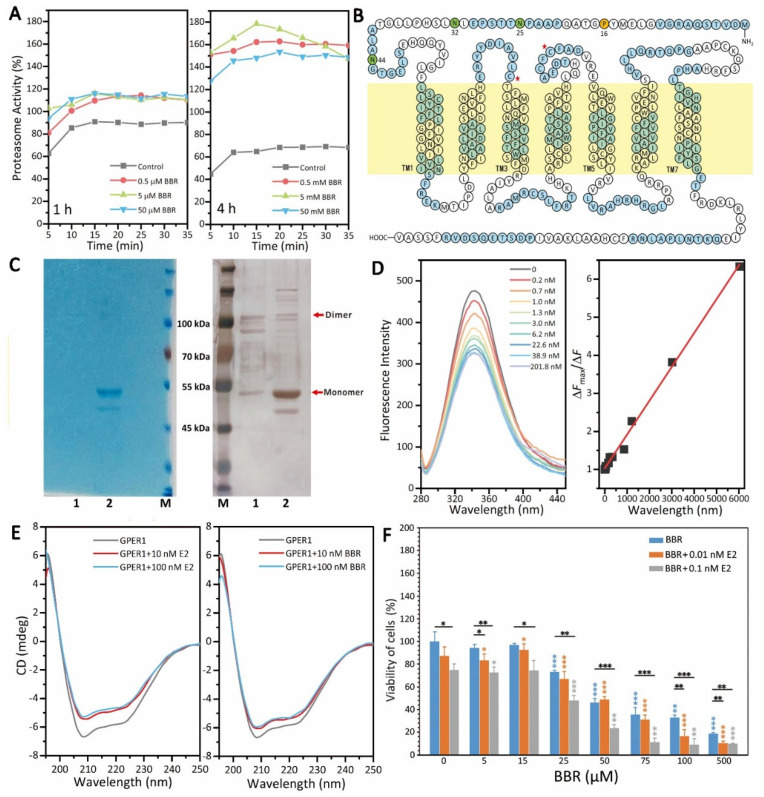
BBR was an agonist of GPER1. (**A**) BBR treatment activated proteasome activity. (**B**) The sequence of human GPER1 is shown. Colors were changed after every ten residues. There was a total of 25,32,44 N-glycosylation sites; 16P-susceptible sites. (**C**) Two collections with size exclusion chromatography were studied using both SDS-PAGE and Western blot. (**D**) The fluorescence intensity of GPER1 decreased with no shift in the maximum fluorescence emission wavelength when the concentration of BBR increased. (**E**) BBR produced a secondary structure change in GPER1 similar to E2. (**F**) BBR inhibited the cell viability of MDA-MB-231 cells in a way that relied on the level of E2. The data are presented as mean ± SEM; *n* = 5. *p* < 0.05 (*), *p* < 0.01 (**), *p* < 0.001 (***).

GPER1 could be hydrolyzed by the 20S proteasome [22]. To clarify the mechanism underlying the upregulation of GPER1, we tested the effect of BBR on the activity of the 20S proteasome. As shown in Figure 3A, BBR treatment activated proteasome activity. Given that BBR modulates the intracellular distribution of GPER1 and regulates the expression of its target genes, we hypothesized that BBR might regulate the function of GPER1 through direct binding. To test the interaction between BBR and GPER1, we constructed a pFastbac1 plasmid encoding the fragment of human GPER1 (Figure 3B) for protein expression and purification. As shown in Figure 3C, the successful expression of human GPER1 was studied by SDS-PAGE and Western blot. As shown in Figure 3D, the fluorescence intensity of GPER1 decreased with no shift of the maximum fluorescence emission wavelength when the concentration of BBR increased. However, the ultraviolet absorption of BBR in the same wavelength range was weak (Supplementary Figure S2), which meant the fluorescence quenching should be attributed to the direct binding of BBR to GPER1. The plot of fluorescence intensity could be fitted to the following equation: Δ*F*/Δ*F*_max_ = [BBR]/([BBR] + *K*_d_), where Δ*F* is the fluorescence intensity change after each injection, Δ*F*_max_ is the maximum fluorescence intensity change, and [BBR] is the concentration of BBR. The plot of the fluorescence intensity vs. BBR concentration was approximately linear within this range. The *K*_d_ of BBR derived from the binding curve was 0.89 nM, indicating that the binding of BBR to GPER1 was very strong. Moreover, BBR changed the secondary structure of GPER1 similar to E2 (Figure 3E), which further proved that BBR promotes the GPER1 pathway as an agonist of GPER1. In addition, the inhibitory effect of BBR on the viability of MDA-MB-231 cells was shown to have a dependence on estrogen levels (Figure 3F), which could also be observed on MDA-MB-468 cells (Supplementary Figure S3). Interestingly, the effects of BBR on MDA-MB-436 cells were influenced by estrogen levels in a very different way (Supplementary Figure S3).

### 2.4. BBR Treatment Reduced Autophagy

**Figure 4:**
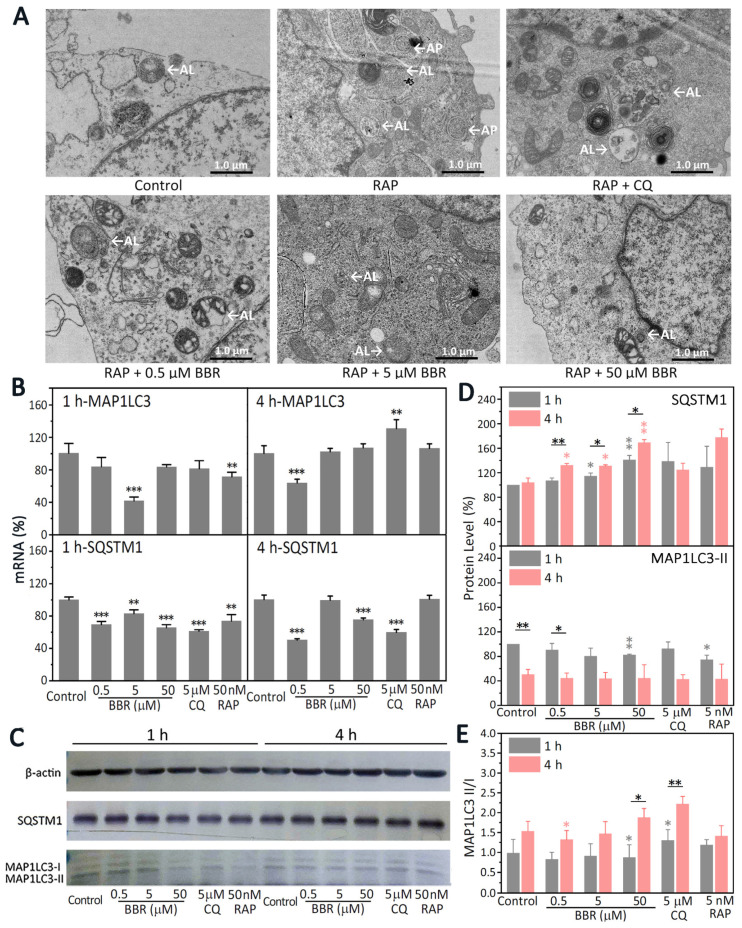
BBR inhibited the autophagy of MDA-MB-231 cells. MDA-MB-231 cells were treated with 0.1% DMSO, 50 μM BBR, 5 μM CQ, or 50 nM RAP for one hour. (**A**) BBR’s effect on the characteristic autophagic structures in MDA-MB-231 cells. Scale bar: 1.0 μM. (**B**) The mRNA expression of SQSTM1 and MAP1LC3 was determined by RT-PCR. GAPDH was set as the reference. The data are presented as mean ± SEM; *n* = 5. *p* < 0.01 (**), *p* < 0.001 (***). (**C**) The protein level of SQSTM1 and MAP1LC3 in MDA-MB-231 cells was detected by Western blot. β-actin was used as the loading control. (**D**) The protein expression levels of SQSTM1 and MAP1LC3-II were calculated relative to the expression of β-actin, *n* = 3. *p* < 0.05 (*), *p* < 0.01 (**). (**E**) The average ratio of MAP1LC3-II/MAP1LC3-I is shown, *n* = 3. *p* < 0.05 (*), *p* < 0.01 (**).

As known autophagy modulators, the effective doses of CQ and RAP for the regulation of autophagy have been reported to be 5 μM and 50 nM, respectively [23,24]. Compared with the control group, we observed via TEM an increase in the accumulation of both autophagosomes (APs) and autolysosomes (ALs) in the cytoplasm of MDA-MB-231 cells after treatment with RAP. Compared with the RAP group, there were fewer Als, and no Aps, observed in the cells of the group with CQ or BBR (Figure 4A).

Autophagy is a dynamic process. To further clarify how BBR influences autophagy in MDA-MB-231 cells, the mRNA and protein levels of MAP1LC3 and SQSTM1 were observed after 1- and 4-h treatment with BBR. As shown in Figure 4B, the inhibitory effects of both 1-h treatment with 5 μM BBR and 4-h treatment with 0.5 μM BBR on the transcription of *MAP1LC3* were extremely significant (*p* < 0.001). The transcription of *SQSTM1* were significantly inhibited in 1-h treatment groups with 0.5 μM BBR (*p* < 0.001), 5 μM BBR (*p* < 0.01), 50 μM BBR (*p* < 0.001), 5 μM CQ (*p* < 0.001), and 50 nM RAP (*p* < 0.01). The inhibitory effects on the transcription of *SQSTM1* in the 4-h treatment groups with 0.5 μM BBR, 50 μM BBR, and 5 μM CQ were extremely significant (*p* < 0.001).

As shown in Figure 4C,D, compared with the control groups, the protein levels of SQSTM1 were significantly promoted by both 5 μM BBR (*p* < 0.05) and 50 μM BBR (*p* < 0.01) in the 1- and 4-h treatment groups. Compared with the 1-h results, the protein levels of SQSTM1 were significantly increased with the 4-h treatment of BBR (0.5 μM, *p* < 0.01; 5 and 50 μM, *p* < 0.05). The protein levels of MAP1LC3-II were decreased after 1-h treatment with 50 μM BBR (*p* < 0.01) and 50 nM RAP (*p* < 0.05). However, compared with the control groups, the protein levels of MAP1LC3-II did not show a distinct change with all the treatments after 4 h, indicating that MAP1LC3-II in all the groups had consumed a lot with time, which might weaken the differences between groups. As shown in Figure 4C,E, the increase in the MAP1LC3-II/MAP1LC3-I ratio in the 4-h treatment with 50 μM BBR was statistically significant (*p* < 0.05), which was similar to that with 5 μM CQ (*p* < 0.01). Considering that the transcription of SQSTM1 was inhibited, the protein levels of SQSTM1 were increased, together with the decrease in ALs and increase in the MAP1LC3-II/MAP1LC3-I ratio. Thus, we believe that BBR could inhibit autophagy in MDA-MB-231.

### 2.5. BBR Induced Co-Localization of GPER1 and MAP1LC3

To further clarify how BBR influences both estrogen signal and autophagy in MDA-MB-231 cells, the intracellular distribution of GPER1, MAP1LC3, RELA, and SQSTM1 was observed after treatment with BBR for 1 min to 4 h.

**Figure 5:**
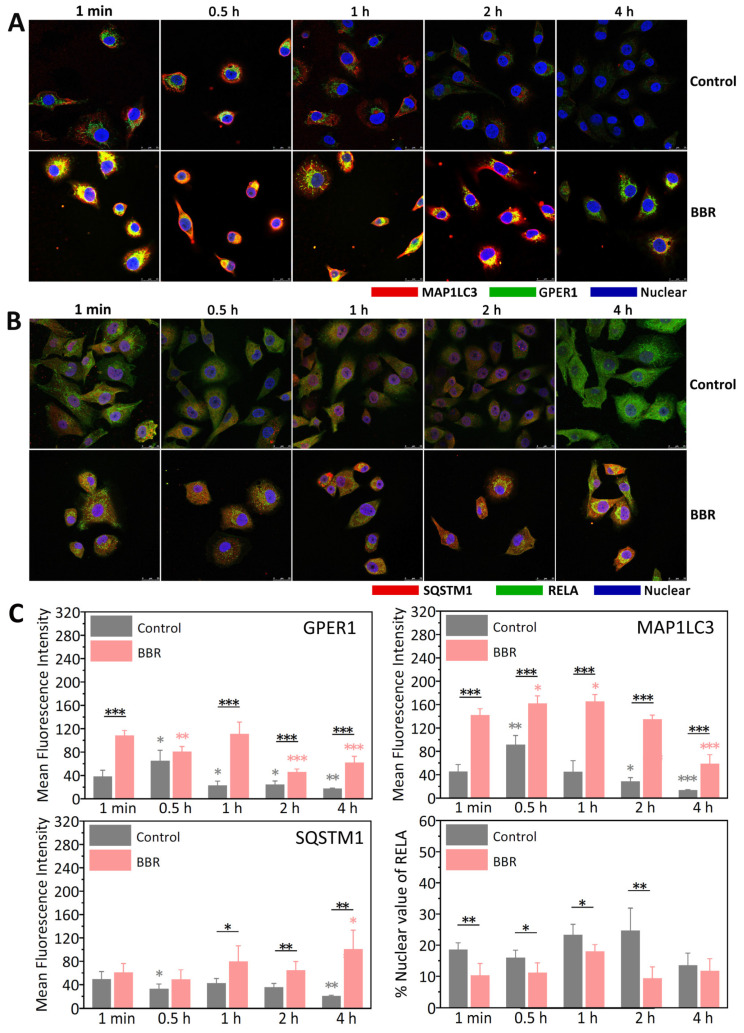
BBR induced a high degree of co-localization of GPER1 and MAP1LC3. MDA-MB-231 cells were treated with 0.1% DMSO and 50 μM BBR for different lengths of time. (**A**) The nucleus, GPER1, and MAP1LC3 were marked with different fluorescent dyes. (**B**) The nucleus, RELA, and SQSTM1 were marked with different fluorescent dyes. (**C**) Mean fluorescence intensity of the proteins (GPER1, MAP1LC3, SQSTM1) and the % nuclear value of RELA were calculated by fluorescence-based quantification, *n* = 4. *p* < 0.05 (*), *p* < 0.01 (**), *p* < 0.001 (***).

As shown in Figure 5A, a high degree co-localization of GPER1 and MAP1LC3 could only be observed in the groups with the treatment of 50 μM BBR. As shown in Figure 5C, comparing the two groups at the same time point, the fluorescence signals of both GPER1 and MAP1LC3 increased obviously with the treatment of 50 μM BBR at almost all the time points (*p* < 0.001). These results further proved the promotional effect of BBR on GPER1 and the co-localization of GPER1 and MAP1LC3 relies highly on the treatment of BBR.

As shown in Figure 5B,C, compared with the control group, the fluorescence signal of SQSTM1 increased clearly with the treatment of 50 μM BBR (1 h, *p* < 0.05; 2 and 4 h, *p* < 0.01). The % Nuclear values were used to measure the nuclear localization of RELA which were significantly decreased with the treatment of 50 μM BBR (0.5 and 1 h, *p* < 0.05; 1 min and 2 h, *p* < 0.01). These results further proved the inhibitory effect of BBR on both autophagy and the nuclear translocation of RELA.

## 3. Discussion

BBR is an isoquinoline alkaloid that has shown multiple cellular effects against the highly aggressive TNBC cell line MDA-MB-231 (Figure 1A–C). However, the exact mechanism remains unclear. The path analysis results based on label-free MS (Supplementary Table S4) revealed that BBR could affect estrogen signaling and some closely related pathways, such as NF-κB, autophagy, and ubiquitin-mediated proteolysis (Figure 1D).

GPER1 is a very important receptor for the regulation of estrogen signaling in MDA-MB-231 cells, which makes it a potential therapeutic target for TNBC treatment [6]. However, the specific modulators for GPER1, which can be used in the clinical treatment of breast cancer, have not appeared [25]. Firstly, there are still many unknowns regarding the subcellular localization and cellular function of GPER1. Secondly, GPER1 belongs to the GPCR membrane protein family and has seven transmembrane structures (Figure 3B), and its N-terminal, C-terminal, and intramembrane loops are all flexible, which makes its expression, purification, and preparation of stable samples difficult. That is perhaps the main reason why there is no significant breakthrough in the research of GPER1 structure, function, and its modulators. After 1 h of treatment, BBR significantly promoted the transcription of *GPER1* in all the BBR groups (Figure 2A). After 4 h, BBR did not affect the transcription of *GPER1* anymore (Figure 2A), but 0.5 and 50 μM BBR could significantly increase the protein levels of GPER1 after 4 h of treatment (Figure 2B–D and Figure 5A,C). The transcription of both CCN2 and RELA was also upregulated by BBR after 1 h of treatment (Figure 2A).

It has been reported that activation of GPER1 can suppress migration and angiogenesis of TNBC via inhibition of NF-κB and interleukin 6 (IL6) signals [5]. Activation of NF-κB is followed by phosphorylation of I kappa B (IKB/IκB) by IKB kinase (IKK) [26]. The phosphorylated IKB is rapidly modified by ubiquitinylation and degraded in proteasome [26]. Then, the nuclear localization sequence of NF-κB can be exposed, which leads to it being transported to the nucleus as the hetero-dimeric active complex to promote the transcription of its dependent gene [27]. Thus, the activity of NF-κB can be determined by detecting whether RELA/p65, the main subunit of NF-κB, could be transferred to the nucleus [28]. The activation of GPER1 can inhibit the phosphorylation of IKBKB/IKK-β and IKB, which rapidly decreases the phosphorylation, nuclear translocation, and activity of NF-κB in human NSCLC cells [27]. Here, BBR was shown to promote both the transcription and the expression of RELA (Figure 2A–C). However, it significantly inhibited the nuclear translocation of RELA according to immunostaining (Figure 2E and Figure 5B,C), which proved the inhibitory effect of BBR on the NF-κB signal and the promotional effect of BBR on GPER1.

To clarify the mechanism underlying the promotional effect of BBR on GPER1, we tested the effect of BBR on the activity of the 20S proteasome, which is a major site for the degradation of both intracellular and plasma membrane-derived GPER1 [22]. BBR has been reported to trigger the proteasomal proteolytic pathway in human renal cancer [29]. Interestingly, BBR also promoted proteasome activity in MDA-MB-231, which excluded the possibility that BBR inhibits the degradation of GPER1 by suppressing proteasome activity (Figure 3A). The promotional effect of BBR on the activity of the 20S proteasome after the 4-h treatment was much stronger than that of the 1-h treatment (Figure 3A). However, the promotional effect of BBR on the protein level of GPER1 was also more significant after the 4-h treatment (Figure 2B,C), which further confirmed that BBR could induce GPER1 signaling in some way.

On the basis of previous work [30,31], we expressed and purified the human GPER1 protein using an insect cell expression system (Figure 3C). Although the protein level of GPER1 obtained here was not enough for high-resolution structure analysis, we still successfully evaluated the in vitro binding of GPER1 with the small molecular BBR and the secondary structure changes of GPER1 with BBR. The results showed that BBR could bind to GPER1 (*K*_d_ = 0.89 nM) and changed the secondary structure of GPER1 similar to E2 (Figure 3D,E), which proved that BBR promotes the GPER1 pathway as an agonist of GPER1, and this was consistent with the dependence of the anti-tumor effect of BBR on E2, shown in Figure 3F.

In cells, proteolysis is achieved by two systems: the ubiquitin–proteasome system (UPS) and autophagy pathways [32]. However, research on the effects of BBR on both of the protein degradation pathways is rare. Autophagy is a multi-step, complex process with the emergence of some key autophagy morphologies that require the coordination of numerous proteins [23,33]. Thus, multiple factors need to be thoroughly considered to determine whether autophagy is inhibited or promoted. Autophagy morphologies can be observed directly by TEM [33]. APs are usually two-layer membrane structures. After APs and lysosomes fuse into ALs, they become a monolayer membrane structure or at least a partially monolayer structure [34]. Compared with the group that was only treated with RAP, there were fewer ALs and no APs observed in the group treated with BBR treatment (Figure 4A). In order to clarify how BBR influences the autophagy of MDA-MB-231 cells, molecular markers of autophagy were detected (Figure 4B,C). The most widely used molecular markers in the study of autophagy are MAP1LC3 and SQSTM1 [24,34]. MAP1LC3-I is lipoificated and transformed into MAP1LC3-II, which then localizes at phagocytic vacuoles and APs [35]. Generally speaking, the levels of both MAP1LC3-II and SQSTM1 decrease during autophagy [36]. As shown in Figure 4E, compared with the 1-h results, the MAP1LC3-II/MAP1LC3-I ratio increased with the 4-h treatment with BBR. The protein levels of SQSTM1 were increased. However, the transcription of SQSTM1 was significantly inhibited (Figure 4B–D). Considering the decrease in ALs together with the increase in the MAP1LC3-II/MAP1LC3-I ratio, we believe that BBR inhibited autophagy in MDA-MB-231, which suppressed the formation of ALs and led to the accumulation of SQSTM1 and the increase in the MAP1LC3-II/MAP1LC3-I ratio.

**Figure 6:**
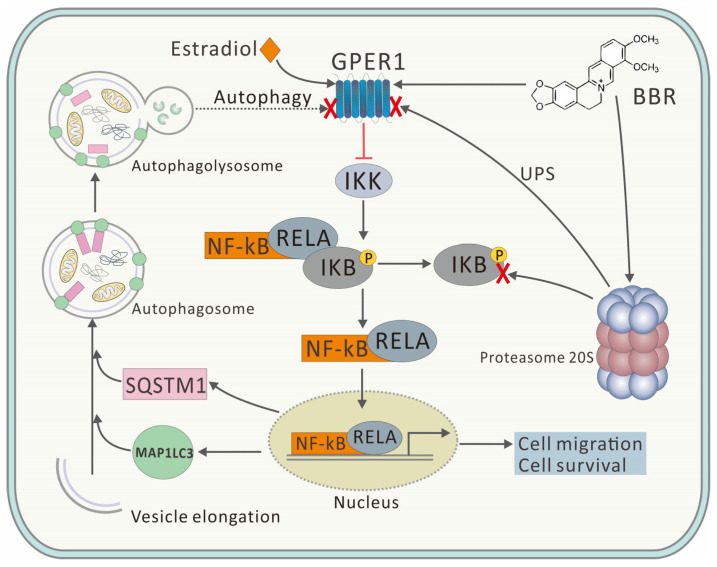
Proposed mechanism of BBR’s inhibitory effect on breast cancer.

The results of the immunofluorescence, shown in Figure 5, further proved this conclusion. BBR induced high degree of co-localization of GPER1 and MAP1LC3, and the high degree of co-localization was only observed in the BBR groups, which further proved the binding of BBR to GPER1. In addition, GPER1 is closely related to MAP1LC3, a key marker protein of autophagy, with the binding of BBR. Correspondingly, both the accumulation of SQSTM1 and the inhibition of the nuclear translocation of RELA could only be observed with the treatment of BBR, which further proved that the inhibitory effect of BBR on autophagy was closely related to the GPER1/NF-κB pathway (Figure 6).

Here, BBR was proved to promote the 20S proteasome and inhibit autophagy (Figure 3A and Figure 4), which might be due to the crosstalk between UPS and the autophagy pathway. Although UPS has been reported to play the major role in degrading GPER1, the inhibitory effect of BBR on autophagy might explain the upregulation of GPER1 and the high degree of co-localization of GPER1 and MAP1LC3 to some extent (Figure 6). This research concerned the effect of BBR on the interplay between estrogen signaling via GPER1, autophagy, and UPS in MDA-MB-231 cells and has furthered our understanding of the function of both BBR and GPER1. However, further research needs to be carried out to verify both the location and the fate of GPER1 during autophagy in MDA-MB-231 cells.

ESR1 is an estrogen receptor that should be absent in TNBCs in theory. However, the expression of ESR1 could restore some factors in MDA-MB-231 cells [37]. It has been reported that immediately following the activation of GPER1, its downstream signaling pathways can ultimately upregulate ESR1 [38]. In addition, the media were replaced with phenol red-free DMEM before all the treatments were conducted, which meant the cells had been under the estrogenic stimulation of the phenol red in the routine culture stage of cells. This might explain the weak expression of ESR1 in all the groups to some extent (Figure 2B).

In summary, our results demonstrated that BBR could promote the 20S proteasome, inhibit autophagy, and induce a high degree of co-localization of GPER1 and MAP1LC3. In addition, BBR could inhibit MDA-MB-231 cells as an agonist of GPER1 by inhibiting the nuclear translocation of RELA, which indicated NF-κB inhibition and anti-cancer effects. This result may demonstrate a new mechanism by which to explain the inhibitory effect of BBR on MDA-MB-231 cells and expand the understanding of the activity of the compound. However, the current research cannot be extrapolated to other TNBC cell lines such as MDA-MB-436. More comparative experiments need to be carried out to explain the difference.

## 4. Materials and Methods

### 4.1. Cell Culture

The breast cancer cell line MDA-MB-231 was obtained from Wuhan Hua Lian Biotechnology Co., Ltd. (Wuhan, China). Both MDA-MB-436 and MDA-MB-468 were obtained from Guangzhou Cellcook Biotech Co., Ltd. (Guangzhou, China). The number of passages of cells used in all described experiments was within 20 passages. MDA-MB-231 cells were authenticated using Short Tandem Repeat (STR) analysis (Genetic Testing Biotechnology, Suzhou, China) in June 2019. Both MDA-MB-436 and MDA-MB-468 cells were authenticated using STR analysis (Guangzhou Cellcook Biotech Co., LTD, Guangzhou, China) in August 2020. Only percent match >80% was regarded as authenticated. The cells were cultured in RPMI-1640 medium with phenol red (HyClone, Logan, UT, USA), which was supplemented with 10% heat-inactivated fetal bovine serum (FBS, Hangzhou Sijiqing Bio-logical Engineering Materials Co., Ltd., Hangzhou, China) in a 37 °C incubator with 5% CO_2_. The media were replaced with phenol red-free DMEM before all the experiments were conducted to avoid estrogenic stimulation of the phenol red. BBR, E2, chloroquine (CQ, an autophagy inhibitor), and rapamycin (RAP, an autophagy promotor) were obtained from (Shanghai Aladdin Bio-Chem Technology Co., LTD, Shanghai, China).

### 4.2. MTT Assay

The effect of BBR on cell viability and proliferation was determined using an MTT (3-[4,5-dimethylthiazol-2-yl]-2,5-diphenyltetrazolium bromide) assay. A total of 100 microliters of cells (8 × 10^4^ cells/mL) were plated in 96-well plates and incubated at 37 °C with 5% CO_2_ overnight. The cells were then exposed to BBR (0, 5, 15, 25, 50, 75, 100, and 500 μM) alone or in combination with E2 (Aladdin, 0.01 or 0.1 nM) for 24 h. After washing once with phosphate buffer saline (PBS), 10 μL of MTT solution (5 mg/mL) was added to each well, and the cells were incubated at 37 °C with 5% CO_2_ for 4 h. After aspirating the culture medium, 100 μL DMSO was added to each well. The optical density (absorbance) at 570 nm was measured with a microplate reader (BioTek Instruments Inc., EON, Winooski, VT, USA). The cell viability of BBR-treated cells was calculated as the percentage of cell viability compared to vehicle control-treated cells, which were arbitrarily assigned as 100% viability. Each independent experiment was repeated five times.

### 4.3. Cell Migration Assay

MDA-MB-231 cells were seeded in a 6-well cell plate at an approximate density of 5 × 10^5^ cells/mL and incubated overnight at 37 °C with 5% CO_2_. Scratches were made through the single layer of cells with a 200 μL tip. The cultures were washed twice with PBS to remove cell debris. At 0 and 24 h post-scratch, the cells within the same fields were observed and photographed. The percentage of wound closure was calculated according to the following formula:

$$Percentage\;of\;wound\;closure=\left(\frac{S_{0\;h}-S_{24\;h}}{S_{0\;h}}\right)\times 100$$

where *S* is the surface area of the wound field.

### 4.4. Label-Free MS Analysis

To explore the effects of BBR on TNBC, serum-starved MDA-MB-231 cells were treated with 0.1% DMSO, 50 μM BBR for 1 h. After being washed with PBS at 4 °C, the cells were harvested, dialyzed, concentrated, and quantified using the BCA method. Samples were adjusted to the same protein concentration, dried, digested with trypsin, and analyzed using mass spectrometry (Thermo Fisher, Orbitrap Fusion, Waltham, MA, USA). The raw file of mass spectrum was searched against the SwissProt protein database (Homo sapiens, 16 July 2019, entries 20,432 proteins) using the MaxQuant software (version 1.6.5.0) with FDR  ≤ 1%. Quantitative analysis was carried out with the IBAQ (intensity-based absolute protein quantification) algorithm [39]. The proteins of BBR group were compared with those of control group. Proteins with similar differences were determined, and we performed a Kyoto Encyclopedia of Genes and Genomes (KEGG) pathway analysis.

### 4.5. Real-Time PCR

MDA-MB-231 cells were treated with 0.1% dimethylsulfoxide (DMSO, Sigma-Aldrich, St. Louis, MO, USA), BBR (0.5, 5, or 50 μM), CQ (5 μM), or RAP (50 nM) for 1 and 4 h. RNA was extracted using TRIzol (Beyotime Biotechnology, R0016, Shanghai, China), according to the manufacturer’s instructions. After the RNA was precipitated and dried, it was dissolved in 50 μL of RNase-free water. cDNA was synthesized from 0.5 μg total RNA from each sample using HiScript II Q RT SuperMix for qPCR (+gDNA wiper) (Nanjing VAZYME Biotech Co., Ltd., R223-01, Nanjing, China). Real-time PCR (RT-PCR) was performed using ChamQ SYBR qPCR Master Mix kit (Nanjing VAZYME Biotech Co., Ltd., Q311-02, Nanjing, China). All the primer sequences used in this study are listed in Supplementary Table S1. The qRT-PCR analysis was performed using the Applied Biosystems (Thermo Fisher, Quant Studio TM 6 Flex, Waltham, MA, USA). The reaction conditions were as follows: 95 °C for 3 min for one cycle followed by 95 °C for 10 s and 60 °C for 30 s for a total of 40 cycles. The differences were calculated according to a ΔΔCt relative quantization method. Glyceraldehyde-3-phosphate dehydrogenase (GAPDH) was used as the control. Each experiment was repeated five times.

### 4.6. Western Blot

To identify the effect of BBR on the expression level of the proteins related to the estrogen signal and autophagy, MDA-MB-231 cells were treated with 0.1% DMSO, BBR (0.5, 5, or 50 μM), CQ (5 μM), or RAP (50 nM) for 1 and 4 h. Then, the treated cells were harvested and lysed directly in 200 μL ice cold lysis buffer containing Tris (50 mM, pH 7.4), 150 mM NaCl, 1% NP-40, and protease inhibitors. The lysates were centrifuged at 10,000× *g* at 4 °C for 10 min. Loading buffer was added in a 1:1 ratio. The samples were collected and separated on a 10% SDS-polyacrylamide gel and transferred to a nitrocellulose filter membrane. The membranes were then incubated overnight with antibodies for MAP1LC3 (1:1000 dilution, Proteintech, Chicago, IL, USA), SQSTM1 (1:2000 dilution, Proteintech, Chicago, IL, USA), GPER1 (1:1000 dilution, Invitrogen, Waltham, MA, USA), CCN2 (1:1000 dilution, Proteintech, Chicago, IL, USA), and RELA (1:2000 dilution, Proteintech, USA), respectively, at 4 °C overnight. The membranes were then washed with Tris-buffered saline (TBS) with Tween 20 (0.1%) and incubated with an alkaline phosphatase-conjugated anti-rabbit or mouse IgG antibody (1:2000) at 37 °C for 2 h. Finally, the proteins were visualized using Enhanced HRP-DAB Chromogenic Substrate Kit (Tiangen, Beijing, China). The antibody β-actin (1:2000 dilution, Proteintech, Chicago, IL, USA) was used as the loading control. Each experiment was repeated in triplicate. Quantification of blots was performed by densitometry.

### 4.7. Immunofluorescence and Confocal Microscopy

MDA-MB-231 cells (500 μL cell suspension) at a density of 1 × 10^5^ cells/mL were added to the central wells of 15-mm laser confocal petri dishes and cultured overnight in a 37 °C incubator with 5% CO_2_. The cells were then treated with 0.1% DMSO, BBR (50 μM), CQ (5 μM), or RAP (50 nM) for one and four hours. The cells were fixed in PBS + 4% formaldehyde for 10 min, permeabilized in PBS + 0.2% Triton X-100 for 10 min, blocked in PBS + 5% BSA for 30 min, and then incubated with the antibody of GPER1 alone or mixed with the antibody of MAP1LC3 or the antibody of RELA alone or mixed with the antibody of SQSTM1 at 4 °C overnight. Both GPER1 and RELA were stained with FITC-conjugated secondary antibody, while both MAP1LC3 and SQSTM1 were stained with Alexa Fluor 594-conjugated secondary antibody for 1 h at room temperature. The nuclei were stained with Hoechst 33258 (Sigma-Aldrich, St. Louis, MO, USA) for 5 min at room temperature. The cells were observed at three different excitation wavelengths (405, 488, or 561 nm) using a laser confocal microscopy (Leica, LCS-SP8-STED, Wetzlar, Germany). The confocal fluorescent images were then used to calculate both the mean fluorescence intensity and the % nuclear value of proteins by fluorescence-based quantification [40].

### 4.8. Transmission Electron Microscope

Six cultures of MDA-MB-231 cells, grown from the same batch, were used for observation of autophagy morphologies. Five of these cultures were treated with 50 nM RAP for 1 h and then treated with BBR (50 μM), CQ (5 μM), or 0.1% DMSO for 1 and 4 h. The remaining culture was treated with 0.1% DMSO for 2 and 5 h as the control, respectively. The cells were collected and fixed with 2.5% glutaraldehyde (pH 7.4) overnight and then incubated with 10 mg/mL citric acid for 2 h. The samples were then dehydrated with acetone. After that, the cells were soaked in 1:1 acetone and epoxy resin for 30 min, embedded with embedding agent, and polymerized at 65 °C. Ultra-thin sections were prepared, stained with uranyl acetate and lead citrate, and observed using a transmission electron microscope (TEM, Hitachi, HT7700, Tokyo, Japan).

### 4.9. Proteasome Activity Assay

It has been reported that the proteasome is a major site for the degradation of GPER1 [22]. To clarify the effect of BBR on 20S proteasome activity, MDA-MB-231 cells were plated onto 6-well plates and maintained in RPMI 1640 medium until 80% confluence. Cells were divided into the control, 0.5 μM BBR, 5 μM BBR, and 50 μM BBR treatment groups for 1 h and 4 h. The cells were washed twice in cold PBS, lysed in 0.5% NP40 for 10 min on ice, and then scraped from wells and centrifuged at 4 °C for 15 min at 13,000 rpm. The supernatant was collected and used to measure the proteasome activity with a Proteasome Activity Assay Kit (Abcam, ab107921, Cambridge, UK). The assay was based on detection of the fluorophore 7-amino-4-methylcoumarin (AMC) after cleavage from the labeled substrate. A total of 20 μg total protein was incubated with a fluorophore-labeled peptide substrate (LLVY-AMC) at 37 °C. The free AMC fluorescence was measured using *E_x_*/*E_m_* = 350/440 nm filter set in a microplate reader fluorometer (Tecan, Männedorf, Switzerland) in the presence/absence of MG132 (a proteasome inhibitor, 10 μM) at 37 °C by dynamic detection for 55 min. An assay buffer without lysate served as blank. Data were calculated by plotting AMC standard curve serial dilutions and normalized by the protein concentrations as mean relative fluorescence units (RFU) (350/440 nm)/μg of total proteins and pmol AMC/μg of total proteins ± SEM (duplicates per experiment).

### 4.10. Protein Expression and Purification

Human GPER1 was expressed in *Sf*9 insect cells for 48 h at 27 °C using recombinant baculovirus. The biomass was harvested by centrifugation at 6000 rpm for 15 min and lysed in RIPA lysis buffer solution (50 mM Tris-HCl, 150 mM NaCl, 1% NP-40, 0.1% SDS), pH = 7.8. Then, 10% glycerol, 1 mM PMSF, and 0.5% (*w*/*v*) n-dodecyl-β-D-maltopyranoside (DDM, Aladdin) were added. After lysis on ice followed by centrifugation, the supernatant was batch bound onto Ni-NTA resin (QIAGEN, Hilden, Germany), and the column was washed with 10 bed volumes of washing buffer containing imidazole, 1% (*w*/*v*) DDM, and 0.2% (*w*/*v*) cholesteryl hemisuccinate (CHS, Aladdin). The collections containing GPER1 were dialyzed and separated by size exclusion chromatography analysis with a superose 6 10/300 GL (GE Life Sciences, Hoevelaken, The Netherlands) column. The eluate presented primarily as monomers, as judged by both SDS-PAGE and Western blotting.

### 4.11. Intrinsic Fluorescence Spectroscopy

Intrinsic fluorescence spectroscopic experiments were performed at 25.0 °C to investigate the interactions of BBR with human GPER1 using a LS-55 luminescence spectrometer (Perkin-Elmer Life Sciences, Shelton, CT, Waltham, MA, USA). An excitation wavelength of 265 nm was used for intrinsic fluorescence measurements, and the emission spectra were recorded between 280 and 450 nm. The excitation and emission slits were both 12.5 nm, and the scan speed was set to 500 nm/min. GPER1 (600 μL) was placed in a 1 mm thermo stated quartz fluorescence cuvette and titrated with BBR with continuous stirring. Both GPER1 and BBR were dissolved in HEPES buffer (10 mM, pH 7.4). The fluorescence of the control buffer titrated with an equivalent amount of BBR was also measured under the same conditions, and the results were used to correct the observed fluorescence of the samples [41]. Each spectrum was scanned in triplicate to acquire the final fluorescence emission spectra.

### 4.12. Ultraviolet Spectroscopy

The ultraviolet spectra of the HEPES buffer (10 mM, pH 7.4) titrated with an equivalent amount of BBR were measured between 280 and 450 nm on a Jasco UV-1700 spectrophotometer (Jasco Corporation, Tokyo, Japan), corresponding to the concentration changes of BBR in the fluorescence experiment.

### 4.13. Circular Dichroism (CD) Spectroscopy

The far-UV CD spectra of GPER1 in HEPES buffer (10 mM, pH 7.4) with no other addition, BBR (0.01 μM or 0.1 μM), or E2 (0.01 μM or 0.1 μM) were measured in the 195–250 nm wavelength range (protein secondary structure) at 25.0 °C on a Chirascan V100 spectrometer (Applied Photophysics, Leatherhead, UK) with 0.1 cm pathlength cylindrical cell. The band width was 1 nm, and the response time was 1 s. Each sample spectrum was corrected by subtraction from the spectrum that was recorded for buffer mixed with an equivalent concentration of the corresponding reagents. The relative change in the α-helical content of GPER1 was represented by the relative change in the molar ellipticity at 222 nm. Each spectrum was an average of three different scans at a scan speed of 100 nm/min.

### 4.14. Statistical Analysis

All the results are presented as the mean ± standard error of mean (SEM). Statistical analyses of the data with equal variances were carried out by one-way or two-way analysis of variance (ANOVA), followed by Tukey’s post hoc test where appropriate. *p* < 0.001 was considered extremely significant. *p* < 0.01 was considered very significant. *p* < 0.05 was considered statistically significant. *p* > 0.05 indicated no statistical difference.

## Supplementary Materials

The following are available online at https://www.mdpi.com/article/10.3390/ijms222111466/s1.
