# Supplementary material for: Berberine Inhibits MDA-MB-231 Cells as an Agonist of G Protein-Coupled Estrogen Receptor 1
Source: Int J Mol Sci. 2021 Oct 24;22(21):11466. doi: 10.3390/ijms222111466 (PMC8583996; doi:10.3390/ijms222111466)
Supplement: Supplementary file 1 [file ijms-22-11466-s001.zip › Figures S1-S3 .pdf]

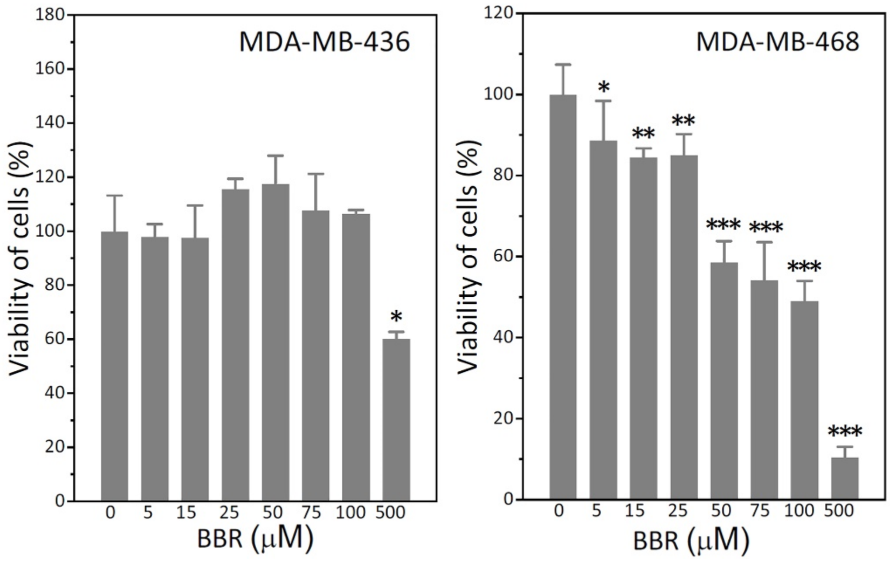

Figure S1. The effect of BBR on the viability of both MDA-MB-436 and MDA-MB-468 cells. The cells were treated with 0.1% DMSO as a vehicle control. The data are presented as mean ± SEM; n = 5. $p < 0.05$ (*), $p < 0.01$ (**), $p < 0.001$ (***).

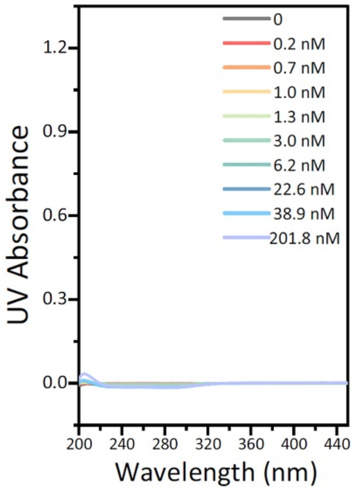

Figure S2. Ultraviolet absorption of BBR.

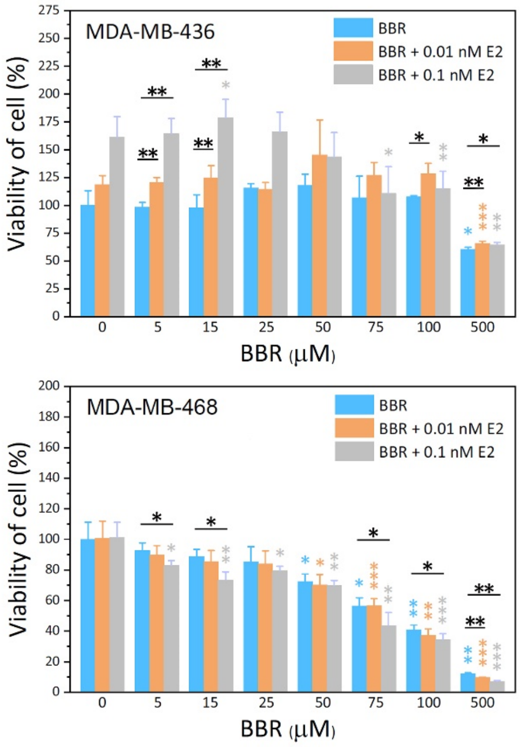

Figure S3. The effect of BBR on the viability of both MDA-MB-436 and MDA-MB-468 cells with the changes of estrogen levels.
