# Supplementary material for: Berberine Inhibits MDA-MB-231 Cells as an Agonist of G Protein-Coupled Estrogen Receptor 1
Source: Int J Mol Sci. 2021 Oct 24;22(21):11466. doi: 10.3390/ijms222111466 (PMC8583996; doi:10.3390/ijms222111466)
Supplement: Supplementary file 1 [file ijms-22-11466-s001.zip › Table S1.pdf]

Table S1. Primer sequences for RT-PCR.

	Primer Sequences (5'–3')
GAPDH	F: TGTTGCCATCAATGACCCCTT
	R: CTCCACGACGTACTCAGCG
SQSTM1	F: GCCAGAGGAACAGATGGAGT
	R: TCCGATTCTGGCATCTGTAG
MAP1LC3	F: AGCAGCATCCAACCAAAATC
	R: CTGTGTCCGTTCACCAACAG
GPER1	F: CTCCAACAGCTGCCTAAACC
	R: TCTACACGGCACTGCTGAAC
RELA	F: AGAAAGAGGACATTGAGGTG
	R: TCGTCTGTATCTGGCAGGTA
